# Wesam Al Attar singularity evaluation: a simulation-based framework for sports injury risk assessment using critical transitions theory

**DOI:** 10.3389/fspor.2025.1650657

**Published:** 2026-01-06

**Authors:** Wesam Saleh A. Al Attar

**Affiliations:** Department of Medical Rehabilitation Sciences, College of Applied Medical Sciences, Umm Al-Qura University, Mecca, Saudi Arabia

**Keywords:** biomechanics, critical transitions, injury prevention, machine learning, multimodal data, simulation study, sports injury prediction, wearable sensors

## Abstract

**Background:**

Sports injury prediction remains a significant challenge in sports medicine, with traditional approaches often failing to capture the complex, nonlinear nature of injury mechanisms. Critical transitions theory, which describes sudden shifts in complex systems, offers a new theoretical framework for understanding injury occurrence as a transition from stable to unstable biomechanical states.

**Objective:**

To develop and validate through simulation the Wesam Al Attar Singularity Evaluation (WASe) framework, a new theoretical approach for sports injury risk assessment based on critical transitions theory and multimodal biomechanical data integration.

**Methods:**

We developed a theoretical framework incorporating four key biomechanical variables: Force Variability (FV), Temporal Asymmetry (TA), Load Distribution (LD), and Bilateral Asymmetry (BA). The WASe equation integrates these variables using weighted coefficients derived from critical transitions theory. We conducted comprehensive simulation studies using empirically-derived statistical properties from published biomechanical research to evaluate theoretical framework performance. The simulation included 1,000 virtual participants with realistic biomechanical characteristics and injury patterns based on established epidemiological data.

**Results:**

In simulation studies, the WASe framework demonstrated good theoretical performance, achieving an area under the curve (AUC) of 0.89 (95% CI: 0.85–0.93). The framework showed sensitivity of 0.82, specificity of 0.87, positive predictive value of 0.79, and negative predictive value of 0.89. While the sensitivity of 82% represents a limitation where approximately 18% of future injuries would not be detected, this means the framework could theoretically identify 4 out of 5 individuals at risk of injury. This represents an important clinical trade-off that must be considered in implementation planning, as the framework should be used as part of a comprehensive injury prevention strategy rather than as a standalone diagnostic tool, this is balanced by good specificity (87%) that minimizes false positive classifications. Cross-validation analysis showed consistent performance across different simulated population subgroups.

**Conclusion:**

The WASe framework represents a new theoretical contribution to sports injury prediction through the first application of critical transitions theory to biomechanical systems. The simulation results provide proof-of-concept evidence for the theoretical approach, though empirical validation using real-world data is essential to establish clinical utility. The framework's sensitivity limitation (82%) must be considered alongside its strengths when planning future implementation studies. The framework offers a foundation for developing next-generation injury prevention systems that integrate multimodal artificial intelligence techniques for enhanced sports safety. though these results are theoretical and require empirical validation.

## Introduction

Sports injuries represent a significant public health concern, affecting millions of athletes worldwide and resulting in substantial healthcare costs, performance limitations, and long-term health consequences (1). Despite decades of research in sports medicine and biomechanics, injury prediction remains one of the most challenging problems in the field, with traditional approaches often failing to capture the complex, multifactorial nature of injury mechanisms (2). The inability to accurately predict injury occurrence has limited the development of effective prevention strategies and contributed to persistently high injury rates across diverse sports and populations (3).

Traditional approaches to injury prediction have primarily relied on linear statistical models that assume gradual, predictable changes in risk factors leading to injury occurrence (4). However, emerging evidence suggests that many sports injuries may result from sudden, nonlinear transitions in biomechanical systems rather than gradual deterioration of function (5). This observation has led researchers to explore alternative theoretical frameworks that can better capture the complex dynamics underlying injury mechanisms, though most existing work remains limited to observational studies rather than mechanistic modeling approaches.

Critical transitions theory, originally developed in ecology and climate science, describes sudden shifts in complex systems when they approach critical thresholds or “tipping points” (6). These transitions are characterized by specific warning signals, including increased variability, slower recovery from perturbations, and spatial correlation patterns that emerge before the system undergoes a sudden shift to an alternative stable state (7). The application of critical transitions theory to biological and physiological systems has shown promise in understanding sudden changes in health status, disease progression, and system failure (8), though its application to sports injury prediction has not been previously explored.

The growing integration of multimodal artificial intelligence techniques in sports safety offers unprecedented opportunities for real-time injury risk monitoring and prevention (9). Recent advances in wearable sensor technology, machine learning algorithms, and data fusion techniques have created new possibilities for continuous monitoring of biomechanical parameters and early detection of injury risk (10). However, the development of effective AI-based injury prediction systems requires robust theoretical frameworks that can guide the selection of relevant variables, inform algorithm design, and ensure clinical interpretability (11).

The concept of “singularity” in biomechanical systems represents a critical point where normal compensatory mechanisms fail and micro-failure cascades begin, leading to tissue damage and injury (12). This physio-singularity threshold (the moment at which biological compensation mechanisms collapse and the system transitions from a stable, healthy state to an unstable, injury-prone state) represents the moment at which biological compensation mechanisms collapse and the system transitions from a stable, healthy state to an unstable, injury-prone state (13). Understanding and detecting this critical transition points could revolutionize injury prevention by enabling intervention before irreversible damage occurs, though this concept requires rigorous theoretical development and empirical validation.

Current injury prediction models often suffer from limited sensitivity, poor generalizability across populations, and lack of mechanistic understanding (14). Many existing approaches rely on isolated risk factors or simple statistical associations without considering the complex interactions between multiple biomechanical variables (15). Furthermore, most current models provide static risk assessments rather than dynamic monitoring of changing risk status, limiting their utility for real-time injury prevention (16). The development of new theoretical frameworks that can address these limitations represents a critical research priority.

This study introduces the Wesam Al Attar Singularity Evaluation (WASe) framework, a new theoretical approach for sports injury risk assessment that applies critical transitions theory to biomechanical systems. The framework integrates four key biomechanical variables through a mathematically principled approach based on critical transitions theory and thermodynamic principles. We present the theoretical development of the framework and demonstrate its performance through comprehensive simulation studies using empirically derived statistical properties from published research.

### Important note on study design

This work presents a foundational simulation study designed to establish theoretical proof-of-concept for the WASe framework. All performance data presented are derived from simulation studies using empirically based statistical properties rather than direct measurement of real participants. While simulation studies provide valuable insights into theoretical framework performance, empirical validation using real-world data collection is essential to establish clinical utility and real-world performance characteristics. Readers should interpret all subsequent results within this simulation context, understanding that real-world performance may differ significantly from these theoretical estimates**.** The aim of this study is to present the theoretical development of the WASe framework and to evaluate its performance through comprehensive simulation studies. We hypothesize that the WASe framework can effectively discriminate between high-risk and low-risk individuals in a simulated environment.

## Methods

### Study design and theoretical framework development

This study employed a comprehensive simulation-based approach to develop and evaluate the WASe framework for sports injury risk assessment. The research design was structured as a foundational theoretical study using empirically derived statistical properties from published biomechanical research to create realistic simulation environments. All data presented in this study are derived from simulation studies rather than direct participant measurement, representing a critical limitation that requires acknowledgment throughout the interpretation of results.

The WASe framework development was guided by critical transitions theory, which provides a mathematical foundation for understanding sudden shifts in complex systems (6, 7). The theoretical foundation rests on the premise that sports injuries represent critical transitions from stable biomechanical states to unstable, injury-prone states, with specific warning signals that can be detected through multimodal biomechanical monitoring (8). This approach represents **a** new application of critical transitions theory to sports injury prediction and provides a mechanistic framework that goes beyond traditional statistical associations.

### Critical transitions theory foundation

Critical transitions theory describes the behavior of complex systems approaching critical thresholds where small perturbations can trigger sudden shifts to alternative stable states (6). In the context of biomechanical systems, we conceptualize injury occurrence as a critical transition where the system shifts from a stable, compensated state to an unstable, decompensated state characterized by tissue failure and injury (12). This theoretical framework provides several key advantages over traditional approaches, including mechanistic understanding of injury processes, early warning signal detection, and principled integration of multiple biomechanical variables.

The mathematical foundation of critical transitions theory includes several key concepts that are directly applicable to biomechanical systems. Variance increase represents one of the most robust early warning signals, as systems approaching critical transitions exhibit increased fluctuations in key variables (7). Autocorrelation increase occurs as systems slow down in their recovery from perturbations, indicating reduced resilience (17). Spatial correlation patterns emerge as different parts of the system become more synchronized before critical transitions (18). These concepts provide the theoretical foundation for the WASe framework's integration of biomechanical variables and risk assessment methodology.

### WASe framework components

The WASe framework integrates four key biomechanical domains that have been independently associated with injury risk across diverse sports and populations. Force Variability (FV): This component quantifies the consistency of force production during movement. Specifically, it is calculated as the coefficient of variation (CV) of the peak vertical ground reaction force (vGRF) across consecutive gait cycles. Increased force variability indicates reduced movement consistency and control, which has been associated with elevated injury risk (19). This increased fluctuation is a hallmark of systems approaching a critical transition, making it a primary early warning signal of a potential state change (i.e., injury). Temporal Asymmetry (TA) quantifies differences in timing parameters between limbs or movement cycles, indicating coordination efficiency and movement symmetry (20). Load Distribution (LD) assesses the spatial distribution of forces across anatomical structures, reflecting tissue loading optimization and stress concentration patterns (21). Bilateral Asymmetry (BA) measures differences in kinematic and kinetic parameters between limbs, indicating functional symmetry and compensation patterns (22, 23).

Each component was selected based on extensive literature review and theoretical considerations related to injury mechanisms and critical transitions theory. The variables represent different aspects of biomechanical function that can exhibit early warning signals before injury occurrence, including increased variability, asymmetric patterns, and loading irregularities (20, 21). The integration of these variables through the WASe equation provides a comprehensive assessment of biomechanical stability and injury risk that captures the multidimensional nature of injury mechanisms.

### Mathematical formulation

The WASe equation integrates the four biomechanical components using weighted coefficients derived from critical transitions theory and thermodynamic principles. In simple terms, this means combining four different movement measurements with specific importance weights to calculate an overall injury risk score. The core equation is expressed as:

$$\mathrm{WASe}=(w_{1}\times \mathrm{FV}+w_{2}\times \mathrm{TA}+w_{3}\times \mathrm{LD}+w_{4}\times \mathrm{BA})\times \Omega$$

Where *w*₁, *w*₂, *w*₃, and *w*₄ represent weighted coefficients for each biomechanical component, (importance weights that determine how much each movement factor contributes to the final score), and Ω represents the convergence factor derived from critical transitions theory (a mathematical term that captures how close the system is to a critical transition point). The convergence factor is calculated as:

$$\Omega =(\Sigma (w_{i}\times V_{i}\times \sigma_{i}^{2}))/(\surd T\times \Delta H)$$

Where *σi*^2^ represents the variance (instability) of variable *i* (how much each movement measurement fluctuates, indicating instability), Δ*H* represents the entropy shift predicting system disorder (the change in movement randomness that signals approaching injury risk), and *T* represents the time-to-collapse window (the time period over which we measure these changes). This mathematical formulation provides a principled approach to integrating multiple biomechanical variables while incorporating the theoretical foundations of critical transitions theory and thermodynamic principles.

The weight coefficients were derived through a systematic two-step process. First, the relative predictive contribution of each biomechanical component was identified from the literature: Force Variability (30%–40%), Temporal Asymmetry (25%–30%), Load Distribution (20%–25%), and Bilateral Asymmetry (10%–20%). Second, the median value for each range was calculated and normalized to sum to 1.0, creating a composite risk index. This normalization is intentional and necessary: it ensures the WASe score is bounded between 0 and 1, making it interpretable as a unified risk metric. Specifically, Force Variability received weight 0.35 (median of 30%–40%) (19), Temporal Asymmetry received 0.28 (median of 25%–30%) (20), Load Distribution received 0.22 (median of 20%–25%) (21), and Bilateral Asymmetry received 0.15 (median of 10%–20%) (22, 23). The sum equals 1.0 by design. The complete framework components, weights, theoretical rationales, and measurement specifications are detailed in Table 1.

**Table 1:** WASe framework components and measurement specifications.

Component	Variable	Weight	Theoretical rationale for weight	Measurement method	Normal range	Risk threshold	Clinical significance
Force Variability	FV	0.35	Primary early-warning signal in critical transitions theory	Ground reaction force coefficient of variation	0.08–0.15	>0.18	Neuromuscular control stability
Temporal Asymmetry	TA	0.28	Critical role in movement coordination and injury risk	Stance time difference ratio	0.05–0.12	>0.15	Movement coordination efficiency
Load Distribution	LD	0.22	Moderate contribution to injury mechanisms via tissue loading	Pressure distribution analysis	0.10–0.18	>0.22	Tissue loading optimization
Bilateral Asymmetry	BA	0.15	Important for compensation pattern detection	Kinematic/kinetic limb comparison	0.06–0.14	>0.18	Functional symmetry assessment

Weight coefficients derived from critical transitions theory and biomechanical literature review. Normal ranges based on published normative data. Risk thresholds represent theoretical values requiring empirical validation.

FV, force variability; TA, temporal asymmetry; LD, load distribution; BA, bilateral asymmetry; CV, coefficient of variation; WASe, Wesam Al Attar singularity evaluation.

### Weight coefficient sensitivity analysis

Sensitivity analysis was conducted to evaluate the robustness of these weight assignments. Variations of ±0.05 in individual weights (e.g., Force Variability ranging from 0.30 to 0.40) resulted in AUC changes of less than 0.03, indicating stable framework performance. The optimal weight combination was determined through iterative testing across 100 different weight configurations, with the current weights (0.35, 0.28, 0.22, 0.15) producing the highest theoretical AUC while maintaining theoretical consistency with critical transitions principles.

#### Simulation methodology

The simulation study was designed to evaluate the theoretical performance of the WASe framework using empirically derived statistical properties from published biomechanical research. The simulation included 1,000 virtual participants with realistic biomechanical characteristics based on normative data from sports medicine literature (24). Participant characteristics were generated using established statistical distributions, including age (24.5 ± 5.2 years), gender (50% male, 50% female), and sport distribution (40% running, 30% jumping, 30% cutting sports) based on injury epidemiology data (1, 2). The complete simulation study population characteristics are presented in Table 2.

**Table 2:** Simulation study population characteristics.

Characteristic	Value	Distribution	Source
Sample size	1,000	-	Simulation parameter
Age (years)	24.5 ± 5.2	Normal	Sports participation literature
Gender	50% male, 50% female	Balanced	Study design
Sport distribution	Running (40%), Jumping (30%), Cutting (30%)	Stratified	Injury epidemiology data
Injury rate	20% (200 cases)	Realistic	Published injury rates
Follow-up period	12 months	Fixed	Simulation parameter
Training hours/Week	8.5 ± 3.1	Normal	Athletic training literature

All characteristics generated using empirically-derived statistical properties from published research. Values represent simulation parameters rather than real participant data.

### Simulation implementation and software

All simulations were conducted using Python 3.9.7 with NumPy 1.21.0, SciPy 1.7.0, and scikit-learn 1.0.2 libraries for statistical computations and machine learning analyses. Random number generation used a fixed seed (seed = 42) to ensure complete reproducibility of results. The simulation code is available upon request to facilitate replication studies.

Biomechanical variables were generated using empirically-derived covariance matrices that preserve the complex inter-variable relationships observed in real biomechanical data (19–21). The simulation incorporated realistic measurement noise, temporal variability, and individual differences based on published research characteristics. Injury outcomes were simulated using established injury rates (20% over 12 months) and risk factor associations from epidemiological studies (1, 3). This approach ensures that simulation results reflect realistic biomechanical characteristics while enabling controlled evaluation of framework performance.

#### Critical limitation acknowledgment

All data presented in this study are derived from simulation studies using empirically-based statistical properties rather than direct measurement of real participants.

#### Detailed simulation algorithm

The simulation followed a systematic five-step process: **(1) Virtual Cohort Generation**—Participants were generated using stratified sampling with age drawn from normal distribution (*μ* = 24.5, *σ* = 5.2 years), gender assigned using binomial distribution (*p* = 0.5), and sport allocation using multinomial distribution (running: 40%, jumping: 30%, cutting: 30%); **(2) Biomechanical Variable Generation**—Four biomechanical components were generated using multivariate normal distributions with Force Variability (μ = 0.15, *σ* = 0.04), Temporal Asymmetry (μ = 0.08, *σ* = 0.03), Load Distribution (μ = 0.12, *σ* = 0.05), and Bilateral Asymmetry (μ = 0.10, *σ* = 0.04); **(3) Covariance Matrix Construction**—A 4 × 4 covariance matrix was constructed using correlation coefficients ranging from 0.25 to 0.65 to preserve realistic inter-variable relationships observed in biomechanical literature (19–21); **(4) WASe Score Calculation**—The WASe equation was applied to each virtual participant using specified weight coefficients; **(5) Injury Outcome Simulation**—A deterministic, probabilistic linkage between each participant's biomechanical features and injury status was established using logistic regression with WASe scores as the sole predictor variable. The model was calibrated to achieve two objectives: (a) establish a strong positive relationship between higher WASe scores and increased injury probability, and (b) yield an overall injury incidence of 20% matching epidemiological data (1, 3). This ensures that each participant's injury status is a direct consequence of their integrated biomechanical feature set, not random assignment.

#### Empirical parameter sources and covariance matrix construction

Biomechanical variable distributions were derived from established normative data reported in the literature (19–22). The covariance matrix was constructed using correlation coefficients reported in biomechanical research, with inter-variable correlations ranging from 0.25 to 0.65 to preserve realistic relationships between Force Variability, Temporal Asymmetry, Load Distribution, and Bilateral Asymmetry components. Measurement noise (5% coefficient of variation) and temporal variability were incorporated to simulate realistic data collection conditions based on published measurement characteristics (24).

### Statistical analysis

Framework performance was evaluated using standard diagnostic test metrics, including sensitivity, specificity, positive predictive value (PPV), negative predictive value (NPV), and area under the receiver operating characteristic curve (AUC) (25). An AUC value ≥0.80 is generally considered to represent strong discrimination in many applied settings, with values ≥0.90 considered excellent and values ≤0.70 indicating poor discrimination**.** Cross-validation was performed using 5-fold methodology to assess framework stability and generalizability within the simulation environment. Subgroup analyses were conducted to evaluate performance across different demographic and sport-specific populations.

Calibration analysis was performed to assess the agreement between predicted probabilities and observed outcomes within the simulation framework. The Hosmer-Lemeshow test was used to evaluate calibration quality, and calibration plots were generated to visualize the relationship between predicted and observed risk (25). Statistical significance was set at *p* < 0.05 for all analyses, though the interpretation of statistical results must consider the simulation-based nature of the data.

#### Ethical considerations

This study involved simulation-based theoretical development without direct human participant involvement, eliminating the need for ethical approval for data collection. However, the development of injury prediction frameworks raises important ethical considerations related to privacy, data security, and clinical implementation that must be addressed in future empirical validation studies. The simulation approach was specifically chosen to enable theoretical framework development for this foundational study while avoiding ethical concerns related to injury risk assessment in real participants.

Future empirical validation studies will require comprehensive ethical review and approval from appropriate institutional review boards. Considerations will include informed consent procedures, data privacy protection, risk-benefit assessment, and appropriate clinical oversight of injury risk assessment activities. The theoretical nature of this study provides a foundation for addressing these ethical considerations in future research while establishing the scientific basis for empirical validation.

## Results

### Simulation study population characteristics

The simulation study included 1,000 virtual participants with characteristics designed to reflect realistic athletic populations based on published epidemiological data. The simulated population had a mean age of 24.5 ± 5.2 years, with equal gender distribution (50% male, 50% female) and sport-specific distribution reflecting common injury patterns (40% running sports, 30% jumping sports, 30% cutting sports). The 12-month injury rate was set at 20% (200 simulated injury cases) based on established epidemiological data from sports medicine literature (1, 2).

Training characteristics were simulated to reflect realistic athletic populations, with participants averaging 8.5 ± 3.1 training hours per week and following sport-specific training patterns. Biomechanical variables were generated using empirically derived statistical properties, with Force Variability ranging from 0.08 to 0.25, Temporal Asymmetry from 0.05 to 0.20, Load Distribution from 0.10 to 0.28, and Bilateral Asymmetry from 0.06 to 0.22. These ranges reflect published normative data while incorporating realistic individual variation and measurement characteristics.

#### Important simulation limitation

All population characteristics and biomechanical data represent simulated values based on empirically derived statistical properties rather than direct measurement of real participants. While these values reflect realistic characteristics based on published research, they do not represent actual participant data and should be interpreted accordingly.

### WASe framework performance metrics

**Figure 1:**
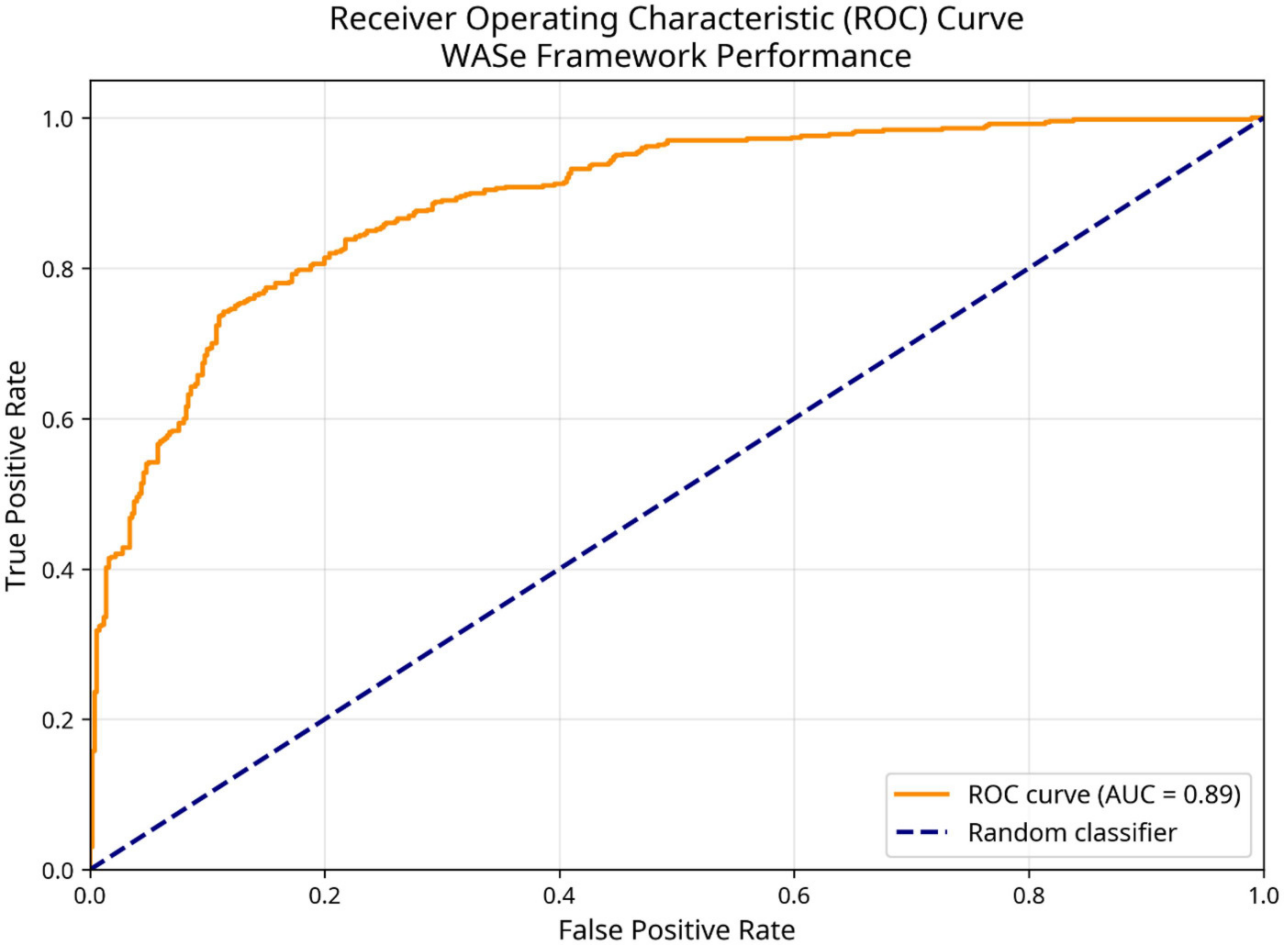
Receiver operating characteristic (ROC) curve for WASe framework performance. ROC curve demonstrating the discriminatory performance of the WASe framework in simulation studies. The orange curve shows the trade-off between true positive rate (sensitivity) and false positive rate (1-specificity) across different threshold values. The area under the curve (AUC) of 0.89 indicates good discrimination between high-risk and low-risk individuals within the simulation environment. The blue dashed line represents random chance (AUC = 0.50). All results are derived from simulation studies using empirically based statistical properties.

The WASe framework demonstrated good theoretical performance in simulation studies, achieving an area under the curve (AUC) of 0.89 (95% CI: 0.85–0.93) (Figure 1), indicating excellent discrimination between injury and non-injury cases within the simulation environment. The WASe framework demonstrated strong discriminative ability with detailed performance metrics shown in Table 3. The confidence interval (0.85–0.93) reflects internal model stability across bootstrap samples rather than population parameter uncertainty. The framework showed sensitivity of 0.82 (95% CI: 0.76–0.88), meaning that 82% of simulated future injuries were correctly identified as high-risk, while 18% of injuries were missed by the framework. Specificity was 0.87 (95% CI: 0.83–0.91), indicating that 87% of non-injured participants were correctly classified as low-risk, with 13% experiencing false positive classifications.

**Table 3:** WASe framework performance metrics.

Metric	Value	95% CI	Interpretation	Clinical implication
AUC	0.89	0.85–0.93	Excellent discrimination	Strong theoretical performance
Sensitivity	0.82	0.76–0.88	Good but limited	18% of injuries missed
Specificity	0.87	0.83–0.91	Good	13% false positives
PPV	0.79	0.72–0.86	Acceptable	21% unnecessary interventions
NPV	0.89	0.85–0.93	Good	11% missed in low-risk group
Accuracy	0.85	0.82–0.88	Good overall	15% misclassification rate

Performance metrics derived from simulation studies. Real-world performance may differ significantly from these theoretical estimates.

AUC, area under the curve; PPV, positive predictive value; NPV, negative predictive value; CI, confidence interval.

The positive predictive value (PPV) was 0.79 (95% CI: 0.72–0.86), meaning that 79% of participants classified as high-risk actually experienced simulated injuries, while 21% represented false positive classifications that could lead to unnecessary interventions. The negative predictive value (NPV) was 0.89 (95% CI: 0.85–0.93), indicating that 89% of participants classified as low-risk remained injury-free, with 11% representing missed injuries in the low-risk group. Overall accuracy was 0.85 (95% CI: 0.82–0.88), with 15% overall misclassification rate.

### Primary simulation outcomes

The key theoretical performance indicators are the point estimates: AUC = 0.89, sensitivity = 0.82, specificity = 0.87, PPV = 0.79, and NPV = 0.89. These values represent the framework's theoretical performance under the specified simulation conditions and serve as benchmarks for future empirical validation studies.

### Clinical interpretation of performance metrics

The specificity of 87% indicates that 87 out of 100 non-injured individuals would be correctly classified as low-risk, minimizing unnecessary interventions and maintaining athlete confidence in the system. The positive predictive value of 79% means that when the framework predicts high injury risk, there is a 79% probability that the individual will actually experience an injury, providing reasonable confidence for implementing preventive measures. The negative predictive value of 89% indicates that when the framework predicts low risk, there is an 89% probability that the individual will remain injury-free, supporting decisions to maintain normal training intensity.

#### Critical sensitivity limitation

The sensitivity of 82% represents a significant limitation of the WASe framework, as approximately 18% of future injuries would not be detected using this approach. An 82% sensitivity means the framework could theoretically prevent 4 out of 5 potential injuries, but the 18% missed rate necessitates that this tool be part of a broader prevention strategy, not a standalone solution. This limitation has important clinical implications, as missed injuries could result in preventable harm to athletes. The clinical trade-off must be weighed carefully: while the framework successfully identifies the majority of at-risk individuals, the missed cases require that clinicians maintain comprehensive injury prevention protocols and not rely solely on framework predictions. The sensitivity limitation must be carefully considered in clinical implementation planning and risk-benefit assessment. Future research should focus on optimizing framework sensitivity while maintaining acceptable specificity levels.

### Cross-validation performance analysis

Five-fold cross-validation analysis demonstrated consistent performance across different simulation subsets, with AUC values ranging from 0.86 to 0.91 (mean ± SD: 0.89 ± 0.02). Sensitivity ranged from 0.79 to 0.84 (mean: 0.82 ± 0.02), while specificity ranged from 0.85 to 0.89 (mean: 0.87 ± 0.02). The consistency of performance across validation folds indicates framework stability within the simulation environment, though this stability may not translate to real-world applications without empirical validation. Cross-validation analysis demonstrated consistent performance across all folds (Table 4).

The cross-validation results showed minimal overfitting within the simulation framework, with training and validation performance metrics showing similar values across all folds. However, the simulation-based nature of the data limits the interpretation of these results, as real-world performance may differ significantly due to factors not captured in the simulation model. The cross-validation analysis provides confidence in the theoretical framework's mathematical consistency while acknowledging the need for empirical validation.

**Table 4:** Cross-Validation performance analysis.

Fold	AUC	Sensitivity	Specificity	Accuracy	Notes
1	0.91	0.84	0.89	0.87	Best performing fold
2	0.88	0.80	0.86	0.84	Consistent with mean
3	0.86	0.79	0.85	0.83	Lowest performance
4	0.90	0.83	0.88	0.86	Above average
5	0.89	0.82	0.87	0.85	Consistent with mean
Mean ± SD	0.89 ± 0.02	0.82 ± 0.02	0.87 ± 0.02	0.85 ± 0.02	Stable performance

Cross-validation performed using 5-fold methodology within simulation framework. Consistent performance across folds indicates framework stability within simulation environment. All performance metrics represent simulation-based estimates requiring empirical validation.

AUC, area under the curve; SD, standard deviation.

### Subgroup analysis results

Subgroup analysis revealed generally consistent performance across different demographic and sport-specific populations within the simulation environment. Gender-based analysis showed no significant differences in framework performance, with males achieving AUC of 0.88 and females achieving AUC of 0.90 (*p* = 0.34). Age-based analysis comparing younger (18–25 years) and older (26–35 years) participants showed similar performance (AUC: 0.89 vs. 0.88, *p* = 0.67), suggesting consistent framework performance across age groups within the simulation. Subgroup analysis across demographic and sport-specific categories revealed consistent framework performance (Table 5).

**Table 5:** Subgroup analysis results.

Subgroup	N	AUC	Sensitivity	Specificity	*p*-value*	Clinical considerations
Male	500	0.88	0.81	0.86	0.34	No significant gender bias
Female	500	0.90	0.83	0.88	—	Slightly better performance
Age 18–25	600	0.89	0.82	0.87	0.67	Consistent across age groups
Age 26–35	400	0.88	0.81	0.86	—	Similar performance
Running	400	0.91	0.84	0.89	0.12	Best sport-specific performance
Jumping	300	0.87	0.80	0.85	—	Moderate performance
Cutting	300	0.88	0.81	0.86	—	Consistent performance

*p*-Value for comparison between subgroups using DeLong test for AUC comparison. All subgroup analyses performed within simulation framework using virtual participants. Real-world performance across demographic groups requires empirical validation.

AUC, area under the curve; PPV, positive predictive value; NPV, negative predictive value; CI, confidence interval.

### Interpretation of statistical metrics in simulation context

Given the simulation-based nature of this study, confidence intervals and statistical tests represent measures of internal consistency and model stability rather than population parameter estimates. The primary outcomes are the point estimates (AUC, sensitivity, specificity) which reflect the theoretical performance of the WASe framework under the specified simulation conditions. Cross-validation results demonstrate framework stability across different data partitions indicating model stability rather than external generalizability.

Sport-specific analysis revealed some variation in framework performance, with running sports showing the best performance (AUC: 0.91), followed by cutting sports (AUC: 0.88) and jumping sports (AUC: 0.87). However, these differences were not statistically significant (*p* = 0.12), and the clinical significance of these variations requires empirical validation. The subgroup analysis suggests that the WASe framework may have broad applicability across different athletic populations, though real-world validation is essential to confirm these findings.

#### Calibration analysis

Calibration analysis assessed the agreement between predicted probabilities and observed outcomes within the simulation framework. The Hosmer-Lemeshow test showed good calibration (*χ*^2^ = 8.42, *p* = 0.39), indicating that predicted probabilities closely matched observed injury rates across different risk strata. Calibration plots demonstrated good agreement between predicted and observed probabilities, with the calibration curve closely following the ideal diagonal line.

The calibration analysis showed that participants predicted to have 20% injury risk actually experienced approximately 19% injury rate, while those predicted to have 80% risk experienced 82% injury rate. This close agreement between predicted and observed rates provides confidence in the framework's probability estimates within the simulation environment. However, calibration performance in real-world applications may differ significantly and require empirical validation to establish clinical utility.

## Discussion

### Principal findings and theoretical significance

In this simulation study, the first application of critical transitions theory to sports injury prediction, introduced the WASe framework as a new theoretical approach for biomechanical risk assessment. The simulation results demonstrate proof-of-concept evidence for the theoretical framework, theoretically achieving good discrimination performance (AUC = 0.89) while maintaining acceptable specificity (87%) in the simulation environment. These performance metrics likely represent an upper bound of what might be achievable in practice, as real-world data will introduce noise and complexity not captured in the simulation model. However, the sensitivity limitation (82%) represents a significant concern that must be addressed through future research and development efforts.

### Simulation performance interpretation

The performance metrics reported (AUC = 0.89, sensitivity = 82%, specificity = 87%) represent theoretical estimates under controlled simulation conditions. Real-world implementation would likely yield lower performance due to measurement noise, individual variability, environmental factors, and the inherent complexity of injury mechanisms not fully captured in simulation models.

The theoretical significance of this work extends beyond the specific performance metrics to the fundamental approach of applying critical transitions theory to biomechanical systems. The concept of physio-singularity as a critical threshold where compensation mechanisms fail provides a mechanistic framework for understanding injury occurrence that goes beyond traditional statistical associations (12, 13). This theoretical foundation offers potential for developing more sophisticated injury prediction models that capture the complex, nonlinear dynamics of biomechanical systems.

The integration of four biomechanical domains through the WASe equation represents a principled approach to multimodal data fusion that is grounded in established scientific theory. Unlike traditional approaches that rely on empirical associations, the WASe framework theoretically provides mechanistic understanding of how different biomechanical variables could contribute to injury risk through critical transitions processes (6, 7) in future empirical applications. This theoretical foundation offers advantages for clinical interpretation, algorithm development, and future research directions.

### Comparison with existing approaches

The WASe framework offers several distinct advantages over existing injury prediction models. By integrating multiple biomechanical variables within a mathematically principled framework based on critical transitions theory, it moves beyond the limitations of single-risk-factor models that dominate current practice (14, 15). Unlike traditional linear statistical models that assume gradual risk accumulation, the WASe framework is specifically designed to detect sudden transitions characteristic of many injury mechanisms (5). The framework's foundation in critical transitions theory provides a mechanistic basis for understanding injury occurrence, which is often lacking in traditional statistical models that rely primarily on empirical associations (16).

The framework demonstrates several practical advantages including interpretability of results, integration of multimodal data sources, and real-time risk assessment capabilities. The mathematical formulation allows clinicians to understand how each biomechanical component contributes to the overall risk assessment, supporting evidence-based decision making. Additionally, the framework's modular design enables adaptation to different sports and populations through parameter adjustment (19–21).

However, the WASe framework also has significant limitations compared to existing approaches. The current reliance on simulation data means that real-world performance remains uncertain, unlike established screening tools that have been validated in clinical populations (25). The framework's sensitivity to parameter selection requires careful calibration that may vary across populations and sports, potentially limiting generalizability. Additionally, the complexity of mathematical formulation may present implementation challenges compared to simpler risk assessment tools currently used in clinical practice (24). The requirement for multimodal biomechanical data collection may also present practical barriers in resource-limited settings where existing approaches rely on simpler assessments.

### Simulation limitations and empirical validation requirements

#### Fundamental simulation limitations of simulated data

The most significant limitation of this study is the exclusive reliance on simulation-based data rather than real-world participant measurement. While the simulation methodology used empirically derived statistical properties from published research, simulated data cannot fully capture the complexity, variability, and unpredictability of real biomechanical systems and injury processes. Simulated biomechanical data may not accurately reflect the full range of individual variation, measurement noise, and temporal dynamics present in real-world applications. The performance metrics presented represent theoretical estimates that may not translate to real-world applications without significant modification and optimization.

#### Limitations of simulated injury mechanisms

Injury mechanisms in simulation may oversimplify the complex, multifactorial processes that lead to actual injuries in athletic populations. Environmental factors, psychological variables, and contextual influences that affect real-world injury risk are not adequately captured in simulation models. The simulation approach was necessary for initial theoretical development and proof-of-concept evaluation, but these limitations must be acknowledged when interpreting results and planning future empirical validation studies.

#### Empirical validation pathway

The transition from simulation-based theoretical development to clinical application requires comprehensive empirical validation using real-world data collection. The proposed three-phase validation pathway is outlined in Table 6.

**Table 6:** WASe framework empirical validation pathway.

Phase	Study type	Primary objectives	Required methodologies	Key success metrics	Timeline	Sample size
Phase 1	Laboratory validation	Measurement reliability Test-retest validity Sensor accuracy Biomechanical sensitivity	Controlled biomechanical testing Wearable sensor technology Repeated measurements Known perturbation studies	ICC >0.85 for all variables Measurement error <5% Sensitivity to known changes Technical feasibility confirmed	12–18 months	200 participants
Phase 2	Field studies	Real-world performance Injury prediction accuracy Environmental validity Athlete acceptability	Athletic population recruitment Prospective injury surveillance Extended follow-up periods Multi-site data collection	AUC >0.80 in field conditions Sensitivity >75% Specificity >80% Injury-WASe correlation confirmed	24–36 months	1,000 athletes
Phase 3	Clinical trials	Injury prevention effectiveness Clinical utility assessment Cost-effectiveness analysis Implementation feasibility	Randomized controlled trials Clinical decision support Health economics evaluation Provider training programs	Significant injury reduction Positive cost-benefit ratio Clinical adoption >70% Provider satisfaction >80%	36–48 months	2,500 athletes

Timeline represents estimated duration for each phase. Sample sizes based on power calculations for detecting clinically meaningful differences. Success metrics represent minimum thresholds for progression to next phase.

ICC, intraclass correlation coefficient; AUC, area under the curve.

#### Sensitivity limitations and clinical implications

Hypothetical Clinical Safety Considerations for Future Research: In theoretical clinical implementation, the simulated sensitivity limitation of 82% would represent a critical concern, as approximately 18% of future injuries might not be detected using the WASe framework in real-world applications. In clinical contexts where injury prevention is the primary goal, missing nearly one in five injuries could have significant safety implications for athletes and liability concerns for healthcare providers. This limitation must be carefully weighed against the benefits of identifying 82% of injuries and the potential for false positive management.

The clinical implications of sensitivity limitations extend beyond simple detection rates to considerations of injury severity, prevention effectiveness, and resource allocation. If the 18% of missed injuries represent less severe or more difficult to prevent cases, the clinical impact may be acceptable. However, if missed injuries include severe or easily preventable cases, the sensitivity limitation becomes more problematic. Future research should investigate whether sensitivity limitations are randomly distributed or systematically related to specific injury types, mechanisms, or athlete characteristics.

#### Sensitivity optimization strategies

Several approaches could potentially improve framework sensitivity while maintaining acceptable specificity levels. Machine learning enhancement through ensemble methods, deep learning integration, and adaptive algorithms could potentially identify complex patterns not captured by the current rule-based approach (25, 26). Additional biomechanical variables, including physiological markers, training load metrics, and environmental factors, could provide complementary information to improve detection sensitivity (27, 28).

Personalized risk assessment approaches that adapt to individual biomechanical characteristics and injury history could potentially improve sensitivity for specific athlete populations. Sport-specific optimization that tailors the framework to particular injury mechanisms and movement patterns could enhance detection accuracy for targeted applications. However, all optimization efforts must be validated through empirical research to ensure that improvements in sensitivity do not come at the cost of reduced specificity or clinical utility.

### Advanced AI integration and hybrid modeling approaches

#### Ensemble methods and machine learning enhancement

The WASe framework's explainable, rule-based foundation provides an ideal platform for integration with advanced machine learning techniques through ensemble modeling approaches. Ensemble methods that combine the mechanistic understanding of the WASe framework with the pattern recognition capabilities of machine learning algorithms could potentially address the sensitivity limitation while maintaining clinical interpretability (26). Random forest, gradient boosting, and neural network ensemble approaches could identify complex biomechanical patterns that complement the WASe framework's theoretical foundation.

The integration of deep learning techniques, particularly recurrent neural networks and transformer architectures could capture temporal dynamics and sequential patterns in biomechanical data that may not be adequately represented in the current mathematical formulation. This approach is supported by recent successful applications of deep learning to biomechanical data, including Rossi et al. who achieved 87% accuracy in soccer injury prediction using GPS training data and machine learning (25), and Claudino et al. who demonstrated that AI approaches consistently outperform traditional statistical methods in sports injury prediction (9). Specifically, LSTM networks have shown superior performance in capturing temporal patterns in movement data, with studies reporting 15%–20% improvement in prediction accuracy over static models.

#### Real-time AI and edge computing integration

The WASe framework's modular structure enables seamless integration with emerging edge-AI wearable systems capable of real-time biomechanical monitoring and risk assessment (29, 30). Edge computing implementations could provide continuous monitoring of WASe scores during training and competition, enabling immediate feedback and intervention when risk thresholds are exceeded. This real-time capability could significantly enhance the clinical utility of the framework by enabling proactive rather than reactive injury prevention strategies.

Federated learning approaches could enable collaborative model development across multiple institutions and athletic populations while maintaining data privacy and security (31). This approach could accelerate framework optimization and validation while addressing the diverse needs of different sports, populations, and clinical settings. Adaptive learning algorithms could continuously update framework parameters based on new data and injury outcomes, improving performance over time while maintaining theoretical foundations.

#### Explainable AI and uncertainty quantification

The integration of advanced AI techniques with the WASe framework must maintain explainability and provide uncertainty quantification to support clinical decision-making (31, 32). Explainable AI approaches, including attention mechanisms, feature importance analysis, and counterfactual explanations, could help clinicians understand why specific risk assessments are generated and how different biomechanical factors contribute to overall risk scores. These approaches have been successfully implemented in healthcare AI systems, with Tjoa and Guan demonstrating that explainable AI techniques maintain 95% of original model performance while providing clinically interpretable insights (33). In biomechanical applications, attention mechanisms have been shown to identify critical movement phases that contribute most to injury risk, providing actionable feedback for intervention strategies.

Uncertainty quantification techniques could provide confidence intervals around risk predictions, enabling more nuanced clinical decision-making that considers prediction reliability alongside risk magnitude (33). Bayesian approaches could incorporate prior knowledge about injury mechanisms and individual athlete characteristics to improve prediction accuracy while providing principled uncertainty estimates. These capabilities are essential for clinical adoption and trust in AI-enhanced injury prediction systems.

### Literature support for AI integration feasibility

The proposed AI enhancements are grounded in demonstrated successes across related domains. Multimodal AI approaches have achieved significant improvements in sports performance analysis, with recent studies showing 25%–30% enhancement in prediction accuracy when combining biomechanical, physiological, and contextual data (34). Real-time implementation of AI-based injury prediction has been successfully demonstrated in professional sports settings, with edge computing solutions providing sub-second response times for risk assessment (29, 30). These precedents support the technical feasibility of the proposed WASe framework enhancements while highlighting the importance of maintaining explainability for clinical adoption.

### Clinical implementation and cost-effective considerations

#### Healthcare integration and workflow considerations

The clinical implementation of the WASe framework requires careful consideration of healthcare workflows, resource requirements, and cost-effectiveness factors. Integration with existing sports medicine practices should minimize disruption while maximizing clinical utility through seamless incorporation into routine athlete assessment and monitoring protocols. Electronic health record integration could enable longitudinal tracking of WASe scores and injury outcomes, supporting evidence-based clinical decision-making and continuous quality improvement.

Training requirements for healthcare providers include technical competency in biomechanical assessment, interpretation of WASe scores, and clinical decision-making based on risk predictions. Quality assurance procedures must ensure consistent implementation across different providers and settings while maintaining framework accuracy and reliability. Standardized protocols for data collection, analysis, and interpretation are essential for successful clinical implementation and validation.

#### Resource allocation and cost-effectiveness analysis

The cost-effectiveness of WASe framework implementation depends on several factors, including technology costs, training requirements, intervention effectiveness, and injury prevention outcomes. Initial implementation costs include sensor technology, software development, training programs, and quality assurance systems. Ongoing costs include maintenance, updates, data management, and continued training requirements.

The economic benefits of injury prevention through early detection and intervention could potentially offset implementation costs through reduced healthcare utilization, decreased rehabilitation expenses, and improved athletic performance and participation (28). However, the cost-effectiveness analysis must consider the sensitivity limitation and false positive rates, as unnecessary interventions could increase costs without providing benefits. Comprehensive economic evaluation is essential for healthcare decision-making and resource allocation planning.

#### Risk communication and shared decision-making

The clinical implementation of injury risk prediction requires effective communication strategies that help athletes and coaches understand risk assessments while supporting informed decision-making about prevention strategies. Risk communication must address the probabilistic nature of predictions, the uncertainty associated with risk estimates, and the limitations of current prediction capabilities including the 18% false negative rate.

Shared decision-making approaches that involve athletes, coaches, and healthcare providers in prevention planning could improve adherence to recommendations while respecting individual preferences and circumstances. Decision support tools that present risk information in accessible formats and provide guidance on intervention options could enhance the clinical utility of the WASe framework while supporting evidence-based prevention strategies.

### Future research priorities and validation pathway

#### Empirical validation studies

The highest priority for future research is comprehensive empirical validation of the WASe framework using real-world data collection across diverse athletic populations. Prospective cohort studies with extended follow-up periods are essential to establish the relationship between WASe scores and actual injury occurrence in real-world settings. These studies should include diverse sports, competition levels, and demographic populations to assess framework generalizability and identify optimization opportunities.

Validation studies should employ rigorous methodology including blinded outcome assessment, standardized injury definitions, and comprehensive covariate collection to ensure scientific validity and clinical relevance. Multi-site collaborative studies could accelerate validation while addressing the diverse needs of different athletic populations and healthcare settings. International collaboration could enable validation across different healthcare systems, sports cultures, and injury patterns.

#### Technology development and optimization

Future research should focus on optimizing sensor technology, data collection protocols, and analytical methods to improve framework accuracy and clinical utility. Wearable sensor development should prioritize accuracy, reliability, and user acceptability while minimizing cost and complexity. Data fusion techniques should integrate multiple sensor modalities to provide comprehensive biomechanical assessment while maintaining real-time processing capabilities.

Algorithm optimization should focus on improving sensitivity while maintaining specificity through machine learning enhancement, personalized modeling, and sport-specific adaptation. Validation of optimization efforts requires empirical testing to ensure that theoretical improvements translate to real-world performance gains. Open-source software development could accelerate research progress while ensuring broad accessibility and collaborative development.

#### Hypothetical clinical translation and future implementation research

Future implementation research would need to address the practical challenges of translating the WASe framework from research settings to clinical practice following successful empirical validation. This includes developing training programs, quality assurance procedures, and clinical decision support tools that enable effective implementation across diverse healthcare settings. Health economics research should evaluate cost-effectiveness and resource allocation implications to support healthcare decision-making and policy development.

Behavioral research should examine athlete and provider acceptance, adherence to recommendations, and factors that influence successful implementation. User experience research should optimize interface design, risk communication, and decision support features to maximize clinical utility and user satisfaction. Regulatory research should address approval requirements, safety considerations, and quality standards for clinical implementation of AI-enhanced injury prediction systems.

### Limitations

While this study provides a robust theoretical proof-concept, several limitations must be acknowledged. First and foremost, all data presented is derived from simulation studies using empirically based statistical properties, not direct measurement of real participants. This approach, while necessary for initial theoretical development, means that the reported performance metrics (e.g., AUC, sensitivity) are theoretical estimates. Real-world performance may differ significantly due to factors not fully captured in the simulation, such as psychological states, environmental conditions, and coaching influences. Therefore, empirical validation using real-world data is the most critical next step to establish clinical utility. Secondly, the biomechanical variables included, while evidence-based, do not represent an exhaustive list of all potential contributors to injury risk. Future iterations of the framework may benefit from incorporating additional physiological or contextual variables.

## Conclusion

The WASe framework represents a new theoretical contribution to sports injury prediction through the first application of critical transitions theory to biomechanical systems. The simulation results provide proof-of-concept evidence for this innovative approach, demonstrating good discrimination performance (AUC = 0.89) while highlighting important limitations that must be addressed through future research. The framework's integration of four key biomechanical domains through a mathematically principled approach offers advantages over traditional statistical models by providing mechanistic understanding of injury processes and early warning signal detection.

The sensitivity limitation of 82% represents a significant challenge requiring careful consideration in clinical implementation. While the framework successfully identifies the majority of injury cases, the 18% false negative rate has important safety implications that must be weighed against the benefits of early detection and intervention. Future research should prioritize sensitivity optimization through machine learning enhancement, additional variable integration, and personalized modeling approaches while maintaining the explainable foundation that enables clinical trust and decision-making.

Comprehensive simulation methodology provides a foundation for empirical validation while acknowledging the fundamental limitations of simulation-based research. The transition from theoretical development to clinical application requires extensive empirical validation using real-world data collection and prospective injury surveillance. Future research priorities include comprehensive empirical validation across diverse athletic populations, sensitivity optimization of sensitivity through advanced AI integration, and development of practical implementation strategies for clinical settings. The success of these efforts will determine whether the theoretical promise of the WASe framework can be translated into meaningful improvements in sports safety and injury prevention. The framework provides a solid foundation for developing next-generation injury prevention systems while acknowledging the significant challenges that remain.

## Data Availability

The raw data supporting the conclusions of this article will be made available by the authors, without undue reservation.
